# Eye movements in scene perception while listening to slow and fast music

**DOI:** 10.16910/jemr.11.2.8

**Published:** 2018-08-11

**Authors:** Marek Franěk, Denis Šefara, Jan Petružálek, Roman Mlejnek, Leon van Noorden

**Affiliations:** University of Hradec Králové, Hradec Králové, Czech Republic; The Prague Conservatoire, Prague, Czech Republic; Ghent University, Ghent, Belgium

**Keywords:** Eye movement, eye tracking, attention, music listening, music tempo, scene perception, background music

## Abstract

To date, there is insufficient knowledge of how visual exploration of outdoor scenes may be influenced by the simultaneous processing of music. Eye movements during viewing various outdoor scenes while listening to music at either a slow or fast tempo or in silence were measured. Significantly shorter fixations were found for viewing urban scenes com-pared with natural scenes, but there was no interaction between the type of scene and the acoustic conditions. The results revealed shorter fixation durations in the silent control condition in the range 30 ms, compared to both music conditions but, in contrast to previ-ous studies, these differences were non-significant. Moreover, we did not find differences in eye movements between music conditions with a slow or fast tempo. It is supposed that the type of musical stimuli, the specific tempo, the specific experimental procedure, and the engagement of participants in listening to background music while processing visual information may be important factors that influence attentional processes, which are mani-fested in eye-movement behavior.

## Introduction

Music can be listened to during many everyday activities. North, Hargreaves, and Hargreaves [1] documented that roughly half of participants’ musical experiences occurred within the home, although approximately 18% of musical experiences occurred in public spaces. Some people listen to music all the time while walking, biking, or driving. In these situations, people should link visual exploration of outdoor scenes with processing of auditory stimuli (i.e., music). Wearing large studio headphones or earbuds that effectively silence all surrounding noise may create various dangerous situations. People need to be able to process information from the surrounding environment to avoid collisions with other people, cars, various objects, or simply for their orientation on a route. To date, there is insufficient knowledge of how this process works in real situations and to what extent the processing of acoustic information may restrict perception of visual information from the surrounding environment.

Heye and Lamont [2], in their study based on subjective reports of participants, propose that listeners create an “auditory bubble” while walking or travelling and listening to music and that they readily switch between inside (music) and outside (surrounding) worlds. In these listening situations, instead of perception of surrounding worlds, the attention might be directed to thoughts, memories, and emotions that are elicited by the music. Music turns people’s attention away from the environment toward inward experiences [3, 4, 5]. In this context, our previous study [6] investigated whether listening to music might mask effects of visual characteristics of a walking route at walking speed. Previous investigations revealed that people walking outdoors spontaneously react to various features of the surrounding environment, which results in a fluctuation of their actual walking speed [7, 8]. It was suggested that the absence of typical changes in walking speed at specific points on the route while listening to music reflects individuals not paying adequate attention to the environmental features of a location. The results revealed that music masked the influence of the surrounding environment only to some extent. Fluctuations in walking speed at specific locations on the route still appeared but were smaller compared to no-music conditions. Thus, the study demonstrated that visual exploration of the surrounding environment may be affected by simultaneously listening to music.

Gaining deeper insight into these processes will require more precise knowledge of how visual exploration of outdoor scenes may be influenced by the simultaneous processing of music. Clearly, analysis of eye movements can provide a better understanding of this problem because research in cognitive neuroscience shows that eye movements are closely linked to visual attention processes [9, 10, 11]. Although there are numerous studies of eye movements and various musical activities in the field of sight-reading research [e.g.,^12, 13, 14, 15^], analysis of eye movements in the perception of outdoor scenes while listening to music has started to be investigated only recently. To date, there have been only two relevant eye-tracking studies [16, 17]; however, they investigated different facets of this problem. Schäfer and Fachner [17] were interested in the attentional shift from visual perception of outdoor scenes to absorption in music, while Maróti and her colleagues [16] investigated changes in eye movement parameters (such as fixation duration, saccade duration, saccade amplitude, and saccade number) in relation to musical tempo.

The study by Schäfer and Fachner [17] provided new information on how eye movements can be influenced by participants simultaneously listening to music while observing outdoor scenes. They examined eye movements of participants viewing a picture (a house by the sea) or a film clip (a videotaped road trip on an empty road through an open landscape) while listening to familiar music, unknown music, or in silence. Popular music, with a tempo of approximately 120 beats per min (bpm), was used as the acoustic stimulus. The authors proposed that listening to music elicits an attentional shift from the outer to the inner world (provoked by absorption in music and the emotions and memories it evoked), resulting in lower eye activity. Their data indicated that music significantly reduces eye-movement activity, with participants exhibiting longer fixations, fewer saccades, and more blinks when they listened to music than when they sat in silence. As a possible explanation, they discussed either an attentional shift from the outer to the inner world or simply a shift of attention from visual to auditory input.

Studies investigating eye movements with different types of auditory stimuli show various results. Coutrot, Guyader, Ionescu, & Caplier [18] investigated the viewing of videos with and without their original soundtracks and found longer fixation durations and longer saccade amplitudes in an audio–visual condition than in a visual one. In the study by Maróti et al. [16], participants listened to drum sequences while viewing various natural scenes. It was found that fixation durations and average intersaccade intervals were longer for the drum sequence conditions compared with the quiet condition. On the contrary, Song, Pellerin, and Granjon [19] reported shorter fixation durations in an audio–visual (sounds, music) condition compared to a visual condition while observing video excerpts. The most recent study by Lange, Pieczykolan, Trukenbrod, and Huestegge [20] showed faster total reading time and faster reading completion time in the music compared silence condition. In contrast, some eye-tracking studies that investigated reading with presentation of auditory stimuli revealed no effect of background music. The study by Cauchard, Cane, and Weger [21] examined the influence of background speech and music on overall reading time (the summed fixation durations) using an eye-movement paradigm and found that background music had no effect on reading process or on eye movements while reading. Similarly, Johansson, Holmqvist, Mossberg, and Lindgren [22] found no significant differences between acoustic conditions (preferred or non-preferred music, noise from a café, silence) for the eye movement measures (fixation duration, saccadic amplitude) during reading.

In investigating eye movements while viewing outdoor scenes together with listening to music, we must take into account that recent research in environmental psychology has revealed that certain environmental features have different effects on eye-fixation behavior. Dupont, Antrop, and Van Eetvelde [23] investigated eye movements while viewing photographs of various landscape types in Belgium that differed in degree of openness and heterogeneity. They found a greater number of fixations in enclosed compared to open landscapes and a greater number of fixations in heterogeneous than in homogeneous landscapes. More importantly, eye-tracking studies by Berto, Massaccesi, and Pasini [24] and Valtchanov and Ellard [25] reported reduced eye-movement activity in terms of number of fixations and eye-travel distance while viewing photographs of natural scenes in contrast to urban scenes. Similar results were found in our recent study [26]. Although the mentioned studies were conducted in a laboratory, similar effects have been shown for viewing real natural environments and various forms of surrogate nature [27]. The difference between viewing natural and built scenes was explained in terms of better perceptual fluency while processing natural scenes compared to urban scenes [28]. Evidence is emerging that fractal complexity, typical of natural scenes, may be a source of perceptual fluency [29]. Fractals capture order and structure by the repetition of similar visual information across multiple scale levels. Thus, walking with music in an urban environment might require more attentional resources, not only because of the necessity to observe traffic and the movement of other pedestrians, both of which are more common in urban than natural environments, but also because a built environment is more difficult to visually process than a natural one [e.g., 30].

To sum, the previous studies [24, 25] found a higher visual exploration while observing urban scenes compared to natural scenes, which is manifested in shorter fixation durations, longer eye travel distance, and a higher number of fixations. It is suggested that processing of urban scenes requires greater visual exploration, compared to natural scenes, because they are more difficult to process. The investigations of processing outdoor scenes while listening to music [16, 17] observed significantly longer fixations while listening to music compared to the no-music condition, which suggests that listening to music while viewing visual images requires additional attentional resources. Thus, there is a question of how these two processes interact with each other, and whether the effect of background music on eye-movement behavior while viewing a scene in comparison to a silent control may be modulated by the type of scene (urban vs. natural).

A further question is whether music tempo might affect the motor system of gaze control. Our study was designed to explore the effect of fast-tempo music in contrast to slow-tempo music and a no-music condition on eye movements. There is a large body of research documenting how certain temporal properties of music (such as rhythm or tempo) induce motor processes in a listener [for review, 31, 32]. The tempo of music can influence the speed of movements in various behavioral domains. For instance, the tempo of music can influence walking speed [e.g., 6, 33, 34] and the speed of various sports activities [35, 36, 37, 38]. Studies from the field of consumer psychology suggest that the tempo of in-store music might influence visual exploration and the consequent process of consumer decision-making [e.g., 39, 40]. Music can also influence mood and arousal level before/during sports performance [41, 42, 43, 44], resulting in an improved performance. Karageorghis, Terry, and Lane [45] developed a conceptual framework for predicting the motivational qualities of music in exercise and sports environments. The authors argue that some types of music motivate bodily movements (namely, in sports), while others do not. To strengthen the effect of tempo, in selecting musical stimuli, we employed Karageorghis’s concept of motivational and non-motivational music [45]. Both types of music were used in our previous study [6], in which participants were asked to walk with music along an urban route. The study showed that fast motivational music accelerated walking speed, whereas slow music decreased walking speed, in contrast to common popular music of diverse tempi.

Because eye-movement behavior is inherently rhythmic, an important question is the possible synchronization between eye movements and a musical beat, which may also have some implications for visual processing of perceived scenes. This line of research is based on the Dynamic Attending Theory [46]. According to this theory, the brain works on the basis of internal neural oscillations that are capable of entraining to external events and targeting attentional energy to expected points in time. In accordance with this theory, it can be expected that if visual attention synchronizes to the rhythm of musical beats, the rhythm of eye movements would align with the musical beats as well [16]. Maróti and her colleagues [16] investigated the effects of music tempo (drum grooves) on the eye movements of participants viewing various natural scenes. They used drum grooves with either 102 or 144 bpm. The results revealed that slow musical beats retarded sampling of visual information. Fixation durations significantly increased at the lower beat frequency compared to the higher beat frequency and to the no-music condition. Although the study revealed modulation of eye movements by a musical beat, it did not find evidence for entrainment. Consistent with Maróti et al.’s [16] findings, Lange et al. [20] used basic musical stimuli (bass drum, synthesized sequence of chords) and reported that increased tempo of the musical stimuli (80–140 bpm) accelerated eye fixations during text reading (but did not affect a visual scanning task). However, the most recent study by Plöchl and Obleser [47] provides contradictory findings. Their participants viewed either a blank gray screen or a scene from a picture book while listening to either isochronous or irregular auditory clicks at different temporal frequencies between 180 and 300 bpm. Auditory stimulation, however, had no significant impact on saccade frequency or timing, either under rhythmic or arrhythmic conditions. Although these mentioned studies used different visual stimuli and auditory stimuli of different tempi and complexity, it can be concluded that the effect of speed of auditory beats on the gaze control motor system needs further clarification.

The aim of the present study was to investigate eye movements while viewing urban or natural scenes while listening to two different types of music, namely fast music that motivates bodily movements and slow music. In view of the above literature, we expect that listening to music while viewing outdoor scenes will increase fixation durations compared to observing the scenes without music. Further, the effect of the environmental features of the observed scene and the interaction between the type of scene and music condition will be examined. We expect fixation durations will decrease while viewing urban vs. natural scenes. This should be the case, because of a lower perceptual fluency of urban scenes compared to natural scenes. We also expect that fixation durations will increase when music is presented. Finally, there is a question of whether music tempo will modulate the speed of eye movements. We predict that increased musical tempo will decrease the duration of fixations.

## Methods

### Participants

Ninety-eight undergraduates participated in the study. The students were young adults aged 18–25 years (*M* age = 21.02 yr., *SD* = 1.30) and included 50 men and 48 women. They were recruited from a range of fields of study (informatics, financial management, tourism) at the University of Hradec Králové. None of the participants had formal musical training. They were compensated by partial course credit. Ethical approval for the experiment was obtained from the Department of Management at the University of Hradec Králové. None of the participants had a self-reported visual disorder or problem. The participants provided written informed consent in which they declared that they were voluntarily participating in the experiment and that they were informed about the experimental procedure.

### Design

A between-subjects design was employed. Participants viewed pictures under three conditions: no music, fast music, and slow music. Two measures of eye movements were selected as the dependent variables: (a) the mean duration of all fixations (in s) within an image and (b) the mean number of fixations within an image. Although the second measure is redundant, it might be interesting for comparison with other studies.

### Eye-Tracking Equipment and Measures

The experiment was controlled by a PC computer with a 1366 × 768 pixel resolution screen and a diagonal of 38 cm. Eye movements were recorded binocularly using a Tobii X2-60 eye tracker with a sampling rate of 60 Hz. The apparatus tracks both eyes simultaneously and automatically determines which eye is left and which is right regardless of head pose and blinking. The binocular data were averaged across eyes. The eye-tracking device was attached under the monitor of a laptop. The device and presentation of stimuli, as well as the data processing, were controlled by the Tobii Studio Version 3.2 software. Two measures of eye movements were used: the mean duration of fixations and the mean number of fixations.

### Materials

**Figure 1.**
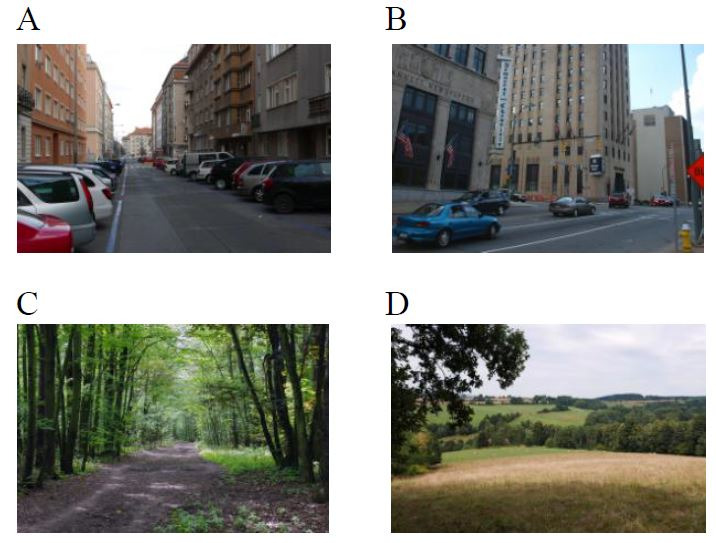
Examples of stimulus material. A, B - urban scenes, C, D - natural scenes.

**Images.** All images used in this study were taken by one of the authors using a digital camera (Nikon D90) with a wide-angle zoom lens. They were composed of images of cities or natural scenery (see Figure 1). All images were transformed to 1152 × 768 pixel resolution using the Adobe Photoshop CS 6 software. Each image was individually optimized. The images had their brightness levels and contrast balanced using the “Auto Levels”, “Auto Contrast”, and “Auto Colors” options in Adobe Photoshop. Twelve images were photographs from cities in the Czech Republic (Beroun, Chlumec nad Cidlinou, Prague, Uherský Brod), Belgium (Brussels) and the United States (Seattle). The urban scenes had a diverse character. Some of them contained high-rise buildings (Seattle), whereas others were streets with typical urban buildings from the 19th century (Brussels, Prague) or streets with low-rise buildings typical of small towns (Beroun, Chlumec nad Cidlinou). The next 12 images with natural scenes were taken in the Czech Republic. They consisted of conifer or deciduous forests, meadows, or ponds. There were no people in any of these images.

**Music.** To prevent the participants from being influenced by the lyrics of the songs, we chose songs in English. Although all participants knew English, it was difficult for non-native speakers to understand the words of the songs. The musical stimuli were borrowed from our previous study [6]. In this study, participants were asked to select and submit two files of different types of music that they liked. The first type of music, motivational music, was characterized as “Music that gives me a strong urge to move in one way or the other”, while the second type of music, non-motivational music, was described as “Nice music, but with no strong urge to move”. Then, they were asked to evaluate the motivational characteristics of these collected musical files, which were made available on a network disk using the Czech version of the Brunel Music Rating Inventory-2 [48]. Based on evaluation using the Brunel Music Rating Inventory-2, musical pieces with the highest motivational character (fast motivational music) and additional pieces with the lowest motivational character (slow, non-motivational music) were selected. For the purposes of the present study, we chose two songs from this selection. The song “One Fine Day” (The Offspring, album Conspiracy of One, 2000) with a tempo of 187 bpm was used as the motivational music. The piece lasted 2 min and 45 s. There was a short half time section (about 20 s) in the song, which repeated three times during the experimental session. The song “Deadmen’s Gun” (Ashtar Command, video game Red Dead Redemption, 2010) with a tempo of 69 bpm was used as the non-motivational music. The piece lasted 3 min and 1 s. By using the VirtualDJ software, a long seamless loop was used in both songs in order to achieve homogenous musical accompaniment as much as possible. The software automatically calculated bpm and then a multiple beat loop (more than a hundred beats long) was created and shifted to the suitable position in the track’s mid-section in order to create the most seamless transition from the loop end to the loop start. The regularity of musical beat was preserved. Participants heard only three transitions between repetitions of the song. The music was presented via headphones and its loudness was adjusted to a comfortable level.

### Procedure

The participants were randomly assigned to a specific condition. There were 17 males and 17 females in the no-music condition. In the fast-music condition, there were 17 males and 17 females, and in the slow-music condition, there were 16 males and 14 females. The following instruction was given to the participants: “You will take part in a study in which you will successively examine a series of images presented on the computer screen. While doing so, you will listen to music on headphones. View each image carefully.” Next, every subject underwent an eye-tracking calibration. Each participant was calibrated once using nine calibration points. The precision of fixations during calibration was evaluated and participants were recalibrated to reach sufficient accuracy. The participants sat approximately 70 cm from the display monitor. The visual angle was 17–22 degrees.

There were 24 images within the experimental session. Twelve images represented urban scenes and another twelve represented natural scenes. The images were presented in a random order. Every trial started with a fixation cross situated in the center of the screen on a light gray background. The initial fixation cross served as a fixation check. The participants had to fixate on the fixation cross for 2 s before the image occurred. The initiated first fixation was not calculated. Each image was displayed for 15 s. The whole experimental session lasted 6 min and 48 s. To simulate real situations when people listen to background music while doing different tasks, in both music conditions the music loop began to play the moment the first slide with the experimental instructions appeared on the computer screen and continued without interruption until the moment when all required tasks were completed. Presentation of stimuli was controlled by Tobii Studio Version 3.2 software.

## Results

### Fixation Durations

The mean fixation durations were calculated for each image and then averaged for type of scene (nature vs. urban) and then averaged across participants (Table 1). There was homogeneity of variances (*p* > 0.05) and covariances (*p* > 0.05), as assessed by Levene's test of homogeneity of variances and Box's M test, respectively. A mixed analysis of variance (ANOVA) was conducted to assess the effects of the type of scene and music condition on the mean fixation durations. The type of scene (nature, urban) was chosen as the within-subject factor, music condition (fast music, slow music, no music) was chosen as the between-subject factor, and the mean fixation duration was the dependent variable. The ANOVA indicated a statistically significant effect of the type of scene, *F*(1, 95) = 28.970, *p* < .001, η^2^ = 0.243, but not a statistically significant effect of music condition (fast music, slow music, no music), *F*(2, 95) = 1.539, *p* = 0.220, η^2^ = 0.031. There was no statistically significant interaction between type of scene and music condition, *F*(2, 95) = 0.258, *p* = 0.773, η^2^ = 0.005. The results showed that the mean fixation duration was significantly longer in the natural scenes than in the urban scenes but did not reveal significant differences among music conditions.

### Number of fixations

The mean number of fixations was calculated for each image, and then averaged for type of scene (nature vs. urban) and then averaged across participants (Table 1). There was homogeneity of variances (*p* > .05) and covariances (*p* > .05), as assessed by Levene's test of homogeneity of variances and Box's M test, respectively. A mixed ANOVA was conducted to assess the effects of the type of scene and music condition on the mean number of fixations. The type of scene (nature, urban) was chosen as the within-subject factor, music condition (fast music, slow music, no music) was chosen as the between-subject factor, and the mean number of fixations was the dependent variable. The ANOVA indicated a statistically significant effect of the type of scene, *F*(1, 95) = 58.761, *p* < .05, η^2^ = 0.382, but not a statistically significant effect of music condition (fast music, slow music, no-music), *F*(2, 95) = 1.973, *p* = 0.145, η^2^ = 0.040. There was no statistically significant interaction between type of scene and music condition, *F*(2, 95) = 0.153, *p* = 0.858, η^2^ = 0.003. The results showed that the number of fixations was significantly greater in urban scenes than in natural scenes but did not reveal significant differences among music conditions.

**Table 1.** Mean scores for the fixation durations in milliseconds and the number of fixations for each experimental condition (no music, slow music, fast music) and the type of scene (urban, nature).

	Urban scenes		Natural scenes	
	Mean	SD	Mean	SD
	Mean fixation durations			
No music	348	59.65	385	99.16
Fast music	369	63.77	420	131.77
Slow music	378	63.70	426	137.09
	Mean number of fixations			
No music	41.85	5.13	38.99	6.83
Fast music	39.75	5.33	36.71	7.11
Slow music	38.20	5.40	36.38	7.44

### Temporal Evolution of Mean Fixation Durations during Stimulus Presentation

In the next step, the temporal evolution of mean fixation durations was analyzed to examine whether the effect of music on fixation durations might change over time during stimulus presentation. We separately calculated results in the three time windows over the course of the visual stimulus presentation: 0–5 s, 5–10 s, and 10–15 s. The mean fixation durations are listed in Table 2 for all time windows separately.

A three-way mixed ANOVA was conducted to assess the effects of the music condition (fast music, slow music, no music), the type of scene (urban, nature) and the time interval (time window) of visual stimulus presentation (0–5 s, 5–10 s, 10–15 s) on fixation durations. There was homogeneity of variances, as assessed by Levene's test for equality of variances (*p* > .05). Greenhouse-Geisser correction was applied where assumption of sphericity was violated as assessed by Mauchly’s test of sphericity.

The ANOVA indicated a statistically significant within-subjects main effect of the type of scene *F*(1, 95) = 28.977, *p* < .001, η2 = 0.234, and a statistically significant within-subjects main effect of the time interval *F*(1.264, 120.077) = 43.809, *p* < .001, η2 = 0.316, but a non-significant between-subjects main effect of music condition *F*(2, 95) = 0.855, *p* = .429, η2 = 0.018. There was a statistically significant two-way interaction between the type of scene and the time interval, *F*(1.406, 133.606) = 12.808, *p* < .001, η2 = .119. There was no statistically significant three-way interaction between the type of scene, music condition, and the time interval, *F*(2.813, 133.606) = 0.440, *p* = .712, η2 = .009. A post hoc analysis with a Bonferroni adjustment showed that the fixation durations were significantly shorter in the 0–5 s interval than in the 5–10 s interval (*p* < .001), in the 5–10 s interval than the 10–15 s interval (*p* < .05), and in the 0–5 s interval than in 10–15 s interval (*p* < .001).

The results showed that the effect of music condition on fixation durations did not change over the course of stimulus presentation. Music condition (fast music, slow music, no music), had no significant effect on fixation durations in the three selected time windows. However, it was observed that fixations durations were gradually extended over the course of stimulus presentation under all experimental conditions. The statistically significant two-way interaction between the type of scene and the time interval shows that the significant effect of the type of the scene appeared at the later stages of the stimulus presentation.

**Table 2.** Mean scores for the fixation durations in milliseconds for particular experimental conditions measured in the three time windows over the course of visual stimulus presentation: 0–5 s, 5–10 s, and 10–15 s.

	Urban scenes		Natural scenes	
	Mean	SD	Mean	SD
	time window 0–5 s			
No music	344	117.38	334	66.76
Slow music	339	56.92	357	93.39
Fast music	358	90.71	393	211.21
Total	347	91.74	361	138.36
	time window 5–10 s			
No music	360	72.75	450	229.74
Slow music	389	82.56	497	246.79
Fast music	399	84.37	491	204.05
Total	382	80.85	479	226.17
	time window 10–15 s			
No music	376	103.61	481	252.08
Slow music	420	142.81	538	364.52
Fast music	432	142.27	521	207.68
Total	409	131.25	513	280.12

## Discussion

This study analyzed eye movements while viewing urban or natural scenes while listening to two different types of music – fast music that motivates bodily movements or slow, non-motivational music – or silence. Significantly shorter fixations were found for viewing urban scenes compared with natural scenes, but we did not find a significant interaction between the type of scene and music condition. The results revealed shorter fixation durations on the range of 30 ms in the no-music condition compared to both music conditions, but these differences were not significant. Moreover, we did not find differences in eye movements between music conditions with either a fast or slow tempo.

While previous studies [16, 17] observed significantly longer fixations while listening to music compared to the no-music condition, which suggests that listening to music while viewing visual images requires attentional resources, we did not succeed in fully replicating these results. Although we did not find significant differences between the music and the no-music condition, we observed a similar trend in both fixation durations and the number of fixations for both the entire viewing time and during the temporal evolution that is consistent with the previous studies [16, 17].

It should be mentioned that the 60Hz sampling rate of our apparatus may be one limitation of this study. While many significant effects on fixation durations are in the range of 30 ms (e.g.,16-19], we did not find significant differences between no music and musical conditions occurring in this range. One possible explanation is that the lower sampling rate of our device might cause higher variability in the data. However, there are also other additional possible explanations.

Schäfer and Fachner [17] used two types of music: self-selected music with the predicted effect of absorption and music from the experimenters, for which absorption was not predicted. However, listening to background music does not necessarily mean that listeners are fully engaged in music listening while performing other activities. Some studies conducted in a service environment (restaurants, shops) showed that people are not often aware of the presence of background music [e.g., 49], particularly if they like the type of music being played. Clearly, listening to background music while viewing scenes does not necessarily recruit a considerable amount of attentional resources to have a conspicuous effect on eye movements. An interest and engagement in the music being played may be an important factor. In Schäfer and Fachner’s [17] study, the participants were asked in advance to bring their favorite music, which may, in general, attract their attention to music in the course of the experiment because they simply may suspect that the experiment has something to do with music perception. Similarly, in Maróti et al.’s [16] study, participants had to perform a tempo discrimination task that may also draw attention to drum sequences during viewing outdoor scenes. In contrast, the experimental procedure used in our study did not necessarily imply that music would be an important part of the experiment.

It should be noted that individual variables might also have an effect on the influence of music on eye-tracking behavior. Although people have individual musical preferences depending on diverse factors [e.g., personality, see 50], we do not expect a confounding effect of musical preference because the musical excerpts used in the experiment were examples of easy-listening popular music. However, individual differences in everyday use of music might potentially have some influence. Chamorro‐Premuzic and Furnham [51] noted that there are three different major uses of music. For some people, music serves mainly for emotional regulation and mood manipulation. Other individuals are characterized by a cognitive approach, which means rational or intellectual processing of music. Finally, background use of music is typical for people who use music as a background for social events, work, or interpersonal interaction. There are also links between these diverse types of music uses and certain personality traits, as found in Chamorro‐Premuzic and Furnham [51]. Clearly, further research should also control for the potential effect of these differences.

Another considerable factor is participants’ musical experience, which may affect their interest in musical or acoustic stimuli while viewing scenes. There is evidence that musicians perceive auditory differences more finely than non-musicians and that they are slightly better at sustained auditory attention than non-musicians [e.g., 52]. Musicians are also better than non-musicians at pre-attentively extracting information out of musically relevant stimuli [53]. Our participants had no formal musical training, and therefore it is unlikely that musical expertise played a confounding role in our study. However, it is worth noting that Schäfer and Fachner’s [17] study did not describe their participants’ musical expertise. In Maróti et al.’s [16] study, some participants were musicians, but the authors did not find any effect of music training. On the other hand, the participants in this experiment listened to simple musical structures, namely, drum sequences. A future research should consider musical expertise as a factor that may play some role.

Although it is usually stated that the average fixation duration for scene perception is between 260 and 330 milliseconds [e.g., 54], the fixation durations found in our experiment were longer. However, it is known that fixation durations for scene perception vary as a function of the task and the characteristics of the scene. For instance, fixation duration is longer for full color photographs than for black-and-white line drawings [55]. They may also be affected by scene luminance [56] and contrast [57]. For instance, in Schäfer and Fachner’s [17] study, average fixation duration values in the no-music condition ranged from 340 to 391 msec.

It is also worth commenting that the duration of visual stimulus presentation might also affect the findings because there might be differences in eye-movement activity between early and late phases within the trial. While in Maróti et al.’s [16] study the participants watched the image for 6 s, in Schäfer and Fachner’s [17] study it was for 45 s, and in our study it was 15 s. However, our analysis of temporal evolution of eye-movement measures within the trial did not reveal significant changes in relation to music condition, though there was an effect of increased fixation duration over image time on screen. It shows that duration of stimulus presentation may also affect the results.

One further question was whether the effect of background music on eye-movement behavior during scene viewing, in comparison to a silent control, may be modulated by the type of scene (urban vs. natural). In accord with previous studies [24, 25, 26], the results showed significantly shorter fixations for viewing urban scenes compared with natural scenes, which is explained in terms of a higher perceptual fluency of natural scenes with respect to ordinary urban scenes. However, our analysis did not reveal any interaction between music and type of scene. This shows that music processing does not interfere with scene processing.

The final question was whether music tempo would modulate the speed of eye-movements. In our study, we did not find differences between the effects of fast and slow music on eye movement. This finding is in contrast with Maróti et al. [16], who reported that the beat frequency of the drum grooves modulated the rate of eye movements, specifically fixation durations, which increased at a lower beat frequency rather than at a higher beat frequency. As we already mentioned, a limitation of our study is that the sampling rate of the eye tracker used did not enable us to measure small differences of approximately 10 ms precisely, as would be expected for beat entrainment. Moreover, it is suggested that the effect of beat frequency on eye movements may be also influenced by the type of musical stimuli and the experimental procedure. In Maróti et al.’s [16] study, the participants listened to the isolated sound of drums; drum grooves might strengthen beat perception. Our stimuli involved ordinary music without a stressed beat. On the other hand, harmonic structure of the pop songs used in our experiment may reinforce metric structure and therefore a beat may be more easily perceived.

However, an alternative explanation is also possible. While Maróti et al. [16] had used music of 102 and 144 bpm to make the effect of musical tempo on eye movements more distinctive, we used tempi with a more salient difference in our experiment. It might be possible that with such a very fast tempo at 187 bpm, participants might extract spontaneously the beat at half of the speed of the music, that is 93 bpm. Moreover, there is a short half time section in the song “One Fine Day”, which can reinforce the 93 bmp feel. If so, the difference between slow music at 69 bpm and fast music with 93 bpm would not be so strong. Interestingly, in our previous experiment [6], in which we explored synchronization between music tempo and walking speed, we found that only one participant synchronized her steps with the beat of the music while listening to a musical piece at a tempo of 187 bmp. Curiously, to synchronize, instead of walking, she was running. However, we did not control bodily synchronization with music in this experiment or the beat extraction process. Clearly, further research should specify the circumstances in which the musical beat may affect eye-movement velocity.

To conclude, the effect of music on eye movements while freely observing outdoor scenes is still not entirely clear. We suggest that the type of stimuli, the specific experimental procedure, and the interest and engagement of participants in listening to background music while processing visual information are important factors that influence attentional processes and the attentional shift from visual to acoustic input, which is manifested in eye-movement behavior.

### Ethics and Conflict of Interest

The authors declare that the contents of the article are in agreement with the ethics described in http://biblio.unibe.ch/portale/elibrary/BOP/jemr/ethics.html and that there is no conflict of interest regarding the publication of this paper.

### Acknowledgements

This research was supported by the Student Specific Research Grant 1/2017 from the Faculty of Informatics and Management at the University of Hradec Králové.
